# Supplementary material for: Diagnostic Yield of TEE in Patients with Cryptogenic Stroke and TIA with Normal TTE: A Systematic Review and Meta-Analysis
Source: Neurol Int. 2021 Dec 1;13(4):659–70. doi: 10.3390/neurolint13040063 (PMC8706810; doi:10.3390/neurolint13040063)

Supplemental file 1.1 The risk of bias assessment.

Study	External validity				Internal validity						
	Was the study's target population a close representation of the national population in relation to relevant variables?	Was the sampling frame a true or close representation of the target population?	Was some form of random selection used to select the sample, OR was a census undertaken?	Was the likelihood of nonresponse bias minimal?	Were data collected directly from the subjects (as opposed to a proxy)?	Was an acceptable case definition used in the study?	Was the study instrument that measured the parameter of interest shown to have validity and reliability?	Was the same mode of data collection used for all subjects?	Was the length of the shortest prevalence period for the parameter of interest appropriate?	Were the numerator(s) and denominator(s) for the parameter of interest appropriate?	Summary item on the overall risk of study bias
Marino et al, 2016	Yes	Yes	Yes	Yes	Yes	Yes	Yes	Yes	Yes	NA	Minor
Gaudron et al, 2014	Yes	Yes	Yes	Yes	Yes	Yes	Yes	Yes	Yes	NA	Minor
Zhang et al, 2012	Yes	Yes	Yes	Yes	Yes	Yes	Yes	Yes	Yes	NA	Minor
Knebel et al, 2009	Yes	Yes	Yes	Yes	Yes	Yes	Yes	Yes	Yes	NA	Minor
de Bruijn et al, 2006	Yes	Yes	Yes	Yes	Yes	Yes	Yes	Yes	Yes	NA	Minor
Blum et al, 2004	Yes	Yes	No	Yes	Yes	Yes	Yes	Yes	Yes	NA	Moderate
de Abreu et al, 2008	Yes	Yes	No	Yes	Yes	Yes	Yes	Yes	Yes	NA	Moderate
Harloff et al, 2005	Yes	Yes	Yes	Yes	Yes	Yes	Yes	Yes	Yes	NA	Minor
Shyu et al, 1993	Yes	Yes	Yes	Yes	Yes	Yes	Yes	Yes	Yes	NA	Minor
Rauth et al, 1996	Yes	Yes	Yes	Yes	Yes	Yes	Yes	Yes	Yes	NA	Minor
Cujec et al, 1991	Yes	Yes	Yes	Yes	Yes	Yes	Yes	Yes	Yes	NA	Minor
Pop et al, 1990	Yes	Yes	Yes	Yes	Yes	Yes	Yes	Yes	Yes	NA	Minor
Pearson et al, 1991	Yes	Yes	No	Yes	Yes	Yes	Yes	Yes	Yes	NA	Moderate
Censori et al, 1998	Yes	Yes	No	Yes	Yes	Yes	Yes	Yes	Yes	NA	Moderate
Retting et al, 2008	Yes	Yes	No	Yes	Yes	Yes	Yes	Yes	Yes	NA	Moderate

Hoy et al., Assessing risk of bias in prevalence studies: modification of an existing tool and evidence of interrater agreement. Journal of Clinical Epidemiology. 65 (2012) 934e939

Supplemental file 1.2: Forest plot of proportion of additional cardiac findings on TEE.

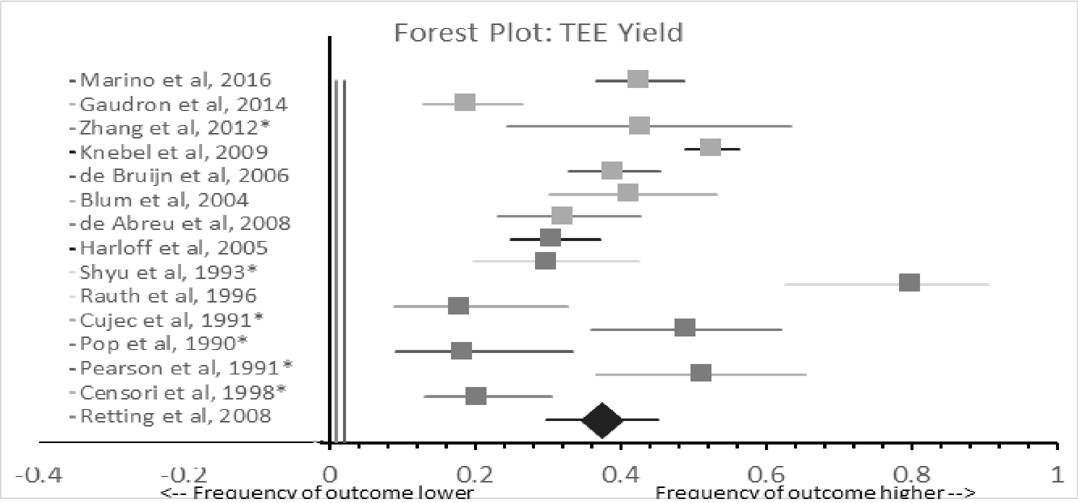

Supplemental file 1.3: Forest plot of proportion of cardiac abnormalities that did change management.

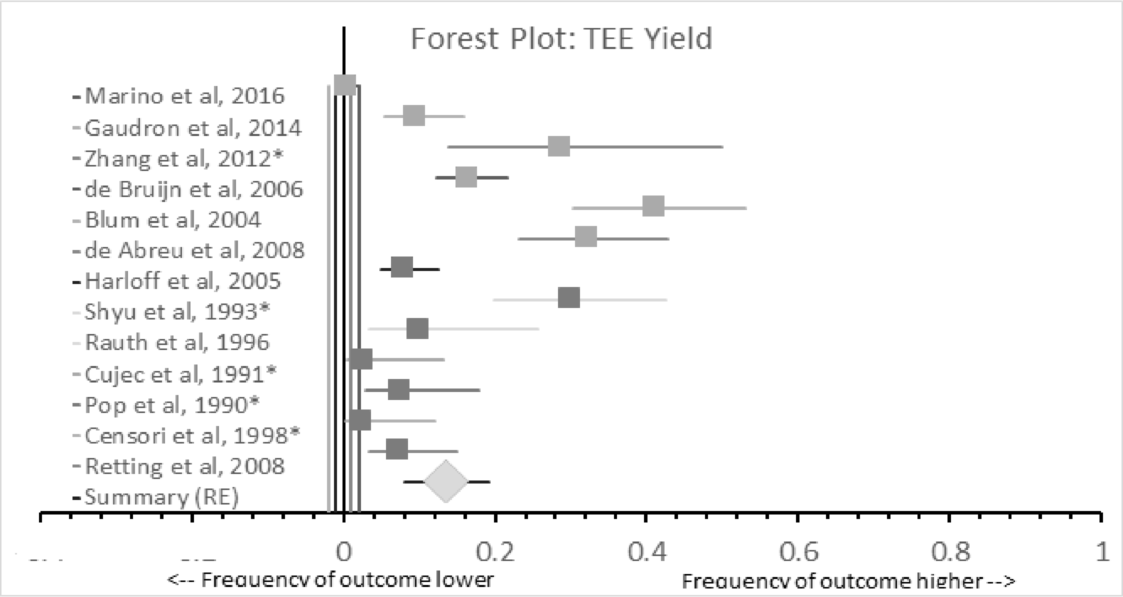

Supplemental file 1.4: Forest plot of proportion of cardiac abnormalities that should change management.

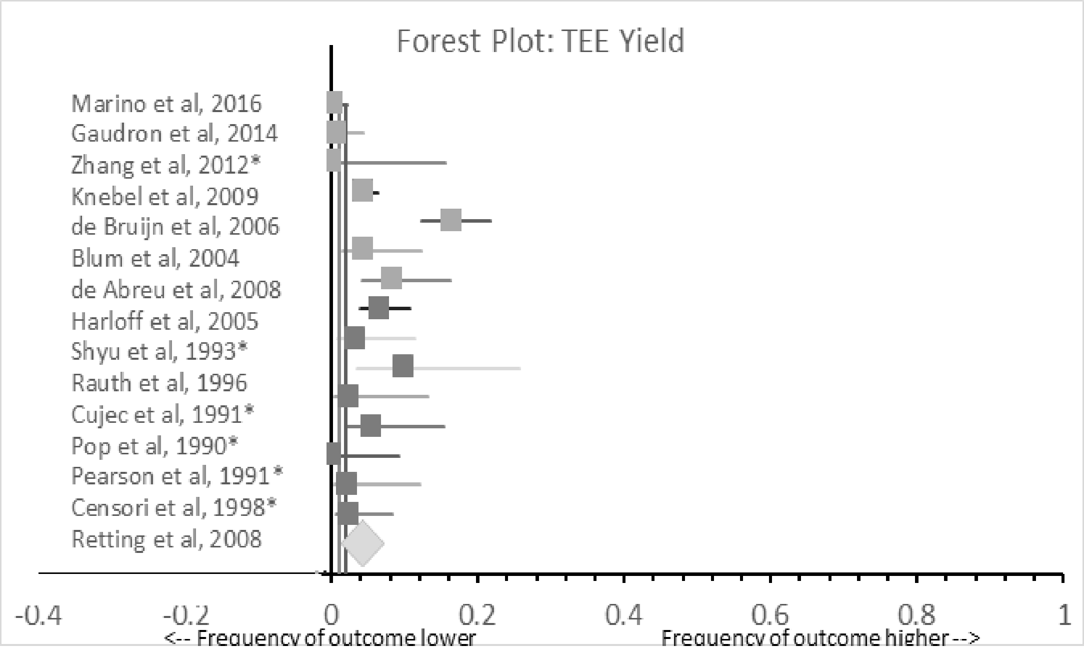

Supplemental file 1.5: Forest plot of proportion of cardiac abnormalities that could change management.

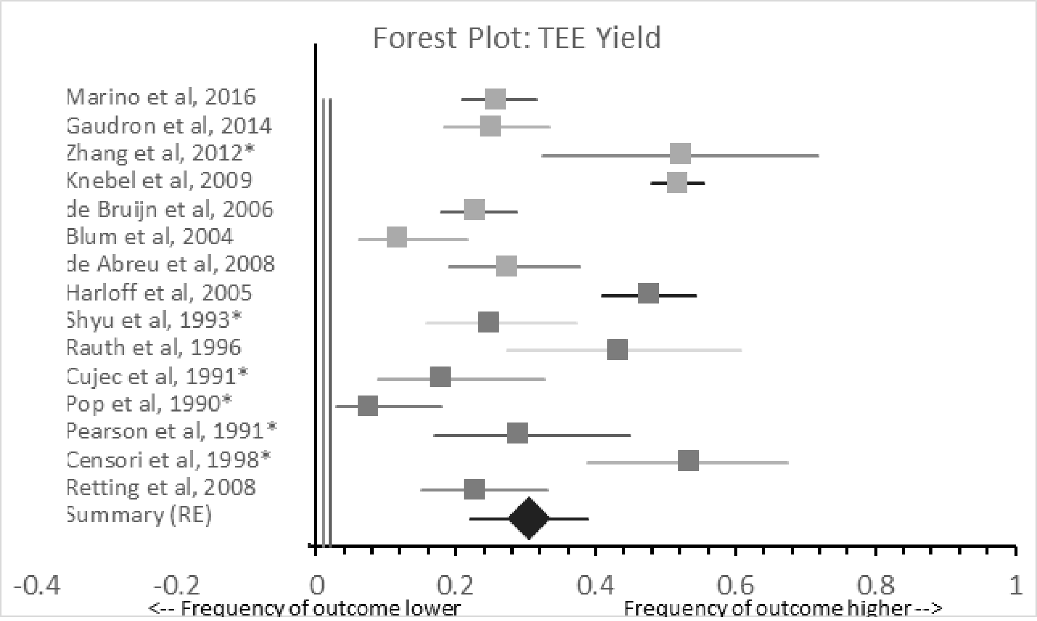

Supplemental file 1.6: Forest plot of proportion of additional cardiac findings on TEE in class II studies.

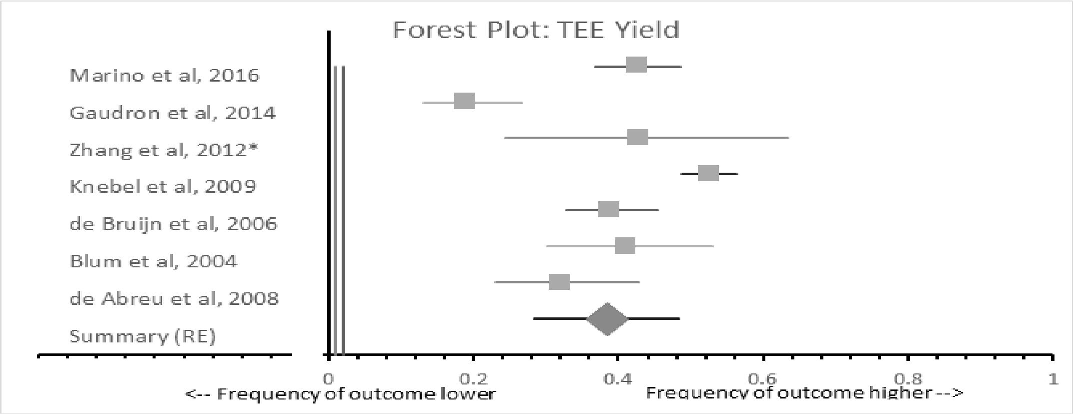

Supplemental file 1.7: Forest plot of proportion of cardiac abnormalities that did change management in class II studies

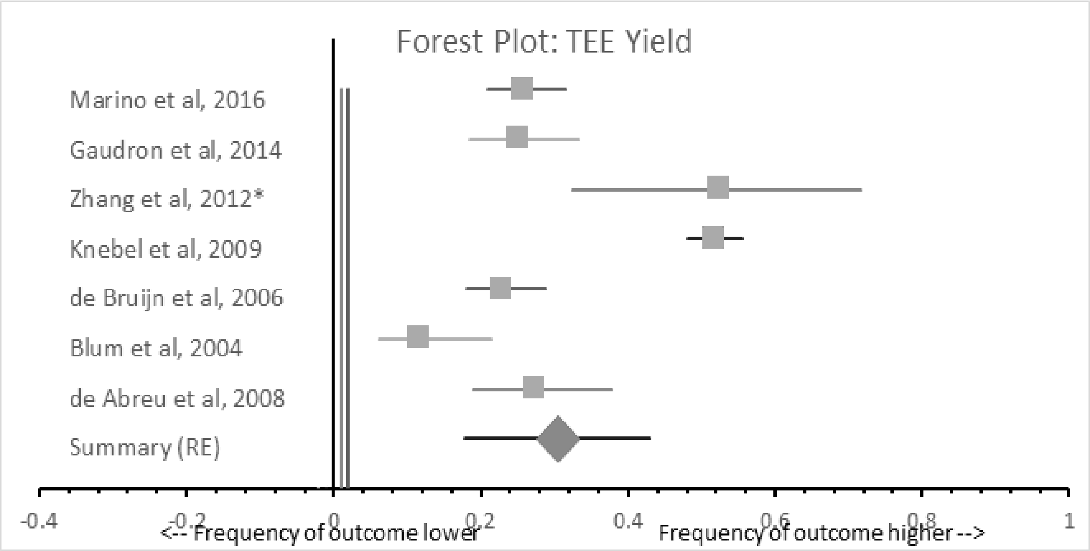

Supplemental file 1.8: Forest plot of proportion of cardiac abnormalities that should change management in class II studies

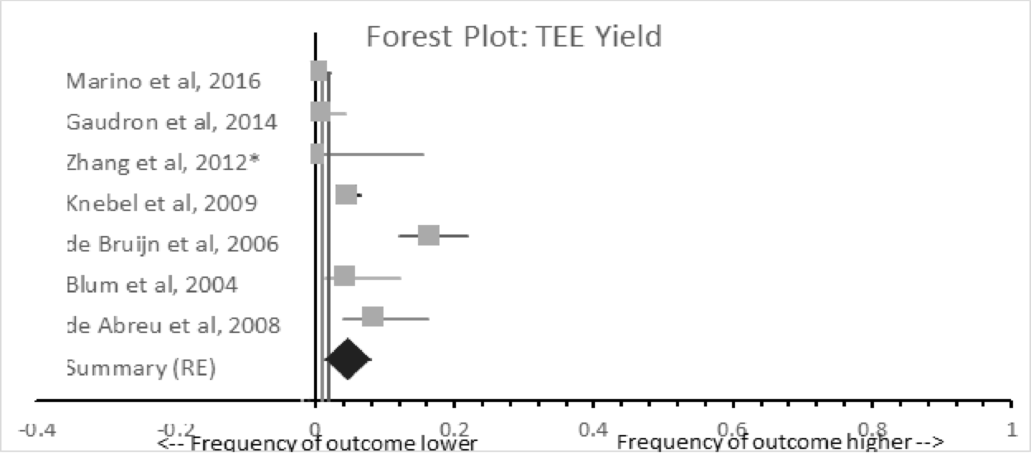

Supplemental file 1.9: Forest plot of proportion of cardiac abnormalities that could change management in class II study.

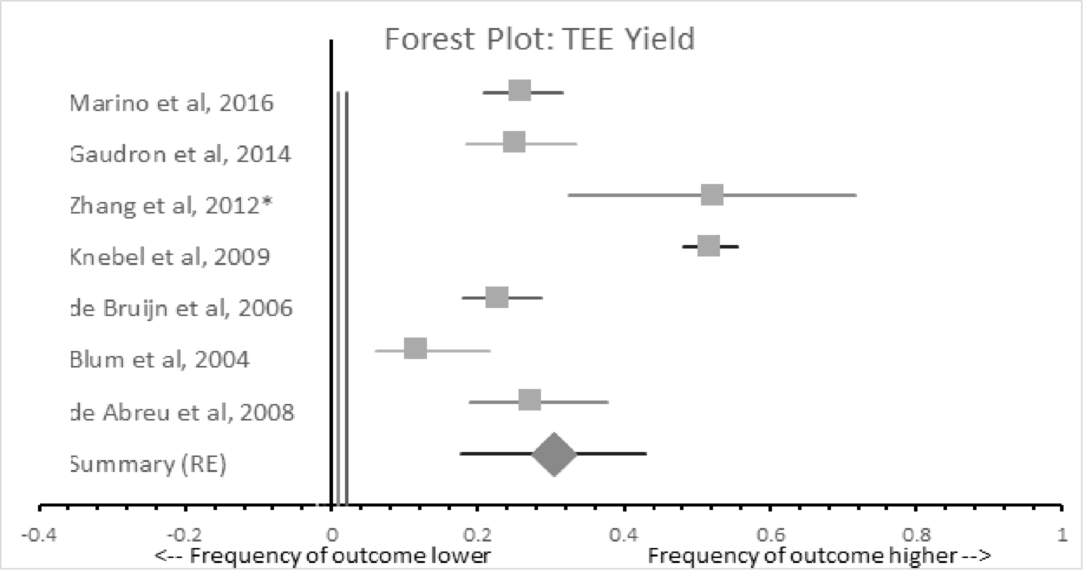

Supplement: Supplementary file 1 [file neurolint-13-00063-s001.zip › neurolint-1263220-supplementary.pdf]
